# Cutaneous metastasis in hypopharyngeal carcinoma: a case report

**DOI:** 10.2478/abm-2024-0006

**Published:** 2024-03-20

**Authors:** Rafia Shahzad, Tooba Anjum, Abu Baker Shahid

**Affiliations:** Department of Radiology, Institute of Nuclear Medicine and Oncology, Lahore 54570, Pakistan; Department of Radiotherapy, Institute of Nuclear Medicine and Oncology, Lahore 54570, Pakistan

**Keywords:** cutaneous metastasis, disease relapse, head and neck squamous cell carcinoma, hypopharyngeal carcinoma, poor prognosis

## Abstract

**Background:**

Occurrence of cutaneous metastasis in hypopharyngeal carcinoma is an extremely rare event reported in the literature, with an incidence of only 0.8%–1.3%. Early diagnosis of cutaneous metastasis would have a positive impact on treatment response and disease prognosis with diagnosis mainly dependent on physical examination and radiological imaging (ultrasonography, computed tomography scan or PET-CT). Palliative care is, however, the mainstay of treatment for cutaneous metastasis.

**Case presentation:**

We report a middle-aged female patient, with known case of hypopharyngeal squamous cell carcinoma, who initially showed partial response to chemoradiotherapy but developed cutaneous nodules in the region of the right axilla and bilateral lateral chest wall posterior to the posterior axillary fold. Excision biopsy of one of these nodules showed metastatic squamous cell carcinoma. The patient was again referred to the Oncology Department of INMOL Hospital and her chemotherapy was planned for cutaneous metastasis.

**Conclusion:**

Being uncommon, the occurrence of cutaneous lesions in a patient with hypopharyngeal carcinoma should prompt detailed evaluation to rule out metastasis. Early detection will help in improving disease prognosis and median survival.

Head and neck squamous cell carcinoma (HNSCC) is the sixth most common cancer in the world [1].

Being uncommon, cutaneous metastasis accounts for only 2% of all skin cancers. In terms of distant cutaneous metastatic lesions, less than 10% of primary tumors metastasize to skin.

In all, 15% of HNSCCs metastasize. Cutaneous metastasis in HNSCC is very rare, with a reported incidence of only 0.8%–1.3%. Of these HNSCCs, carcinoma of the hypopharynx constitutes a small subgroup with extremely rare incidence of cutaneous metastasis; the most common metastatic sites are the liver, lung, and bone.

Occurrence of cutaneous metastasis advocates advanced staging, poor prognosis, treatment failure/disease relapse, and median survival of 7–8 months. Clinically, cutaneous metastasis has a wide spectrum of presentation, thus making it difficult to diagnose at the earliest. Hence, any new cutaneous lesion in a cancer patient should be evaluated in detail to rule out metastasis. In about 6.4%–7.8% of cases, cutaneous meta-stasis precedes metastasis at other distant sites. Out of less than 10% of cutaneous metastasis that arises from head and neck malignancies, most have their origin from squamous cell carcinoma. So here, cutaneous metastasis serves as a clue in predicting the tissue of origin of tumor if primary tumor is unknown.

The majority of cutaneous metastasis occurs in the proximity of the primary tumor site. The scalp and chest wall are the common cutaneous metastatic sites in the case of head and neck malignancies, mostly by means of dermal lymphatic. Diagnosis of cutaneous metastasis depends on physical examination and imaging, which mainly include ultrasonography (USG) and computed tomography (CT) scan to rule out loco-regional or distant involvement and to assess response to treatment. Some authors suggest including dermatological examination in the follow-up of HNSCC patients, as early diagnosis can influence the diagnostic and therapeutic goals [2].

We obtained written informed consent from the patient to publish the present case report and associated images.

## Case presentation

We report a patient with squamous cell carcinoma of the hypopharyngeal region and an unusual cutaneous metastasis as the first indication of disease relapse.

A 45-year female patient presented to the Oncology Out-patient Department at the Institute of Nuclear Medicine and Oncology with the complaint of difficulty in swallowing solids and liquids and odynophagia for the past 18 months. The symptoms progressed with time and were associated with vomiting and weight loss at the time of presentation. Her past medical and family history was unremarkable. She had used only analgesics for her symptoms.

On examination, she was a short lean lady with BMI of 18.2 kg/m^2^ (mild thinness), weight = 42 kg, and height = 1.52 m. She was pale. There was no history of tobacco or alcohol intake, and no previous history of head and neck cancer or radiotherapy in the head and neck region.

**Figure 1.**
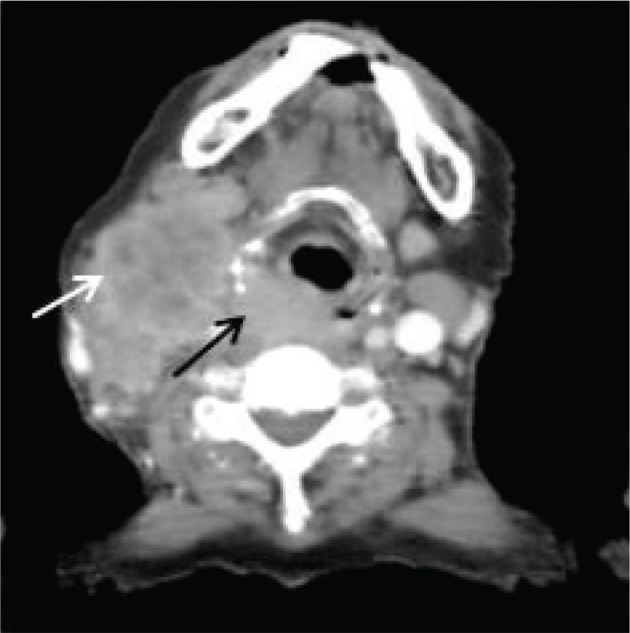
Contrast-enhanced CT scan image through the region of hypopharynx showing a soft tissue density enhancing mass, primarily involving the right side obliterating the right piriform sinus (black arrow). It was extending inferiorly to involve the proximal esophagus. There is also associated right deep cervical confluent nodal mass (white arrow). CT, computed tomography.

CT scan of the face, neck, and chest region following IV contrast administration was performed in the Radiology Department at INMOL Hospital on June 18, 2021 that revealed a soft tissue density enhancing mass measuring 4.8 × 2.9 cm in the region of hypopharynx, primarily involving the right side obliterating the right piriform sinus extending inferiorly to involve proximal esophagus (**Figure 1**). There was bilateral deep cervical and supra-clavicular lymphadenopathy with the largest right deep cervical confluent nodal mass measuring 5.8 × 4.2 cm. There was partial erosion of the thyroid and adjacent cricoid cartilage. The thyroid and salivary glands were unremarkable, no evidence of mediastinal or hilar lymph-adenopathy, and no evidence of pulmonary metastasis.

Histopathological analysis of the hypopharyngeal mass revealed moderately differentiated squamous cell carcinoma.

The patient received 4 cycles of Cisplatin (40 mg in 500 cc of normal saline) and Gemcitabine (1 g) chemotherapy followed by external beam radiation to the hypopharyngeal region. The radiation doses were 70 Gy to high-risk volume in 33 fractions, 60 Gy to intermediate-risk volume in 33 fractions, and 54 Gy to low-risk volume in 33 fractions. The proposed treatment was completed on November 23, 2021. The end of treatment CT scan of the face and neck following the administration of I/V contrast showed interval regression in previously noted hypopharyngeal mass, bilateral deep cervical, and supra-clavicular lymphadenopathy suggestive of partial response to therapy.

**Figure 2.**
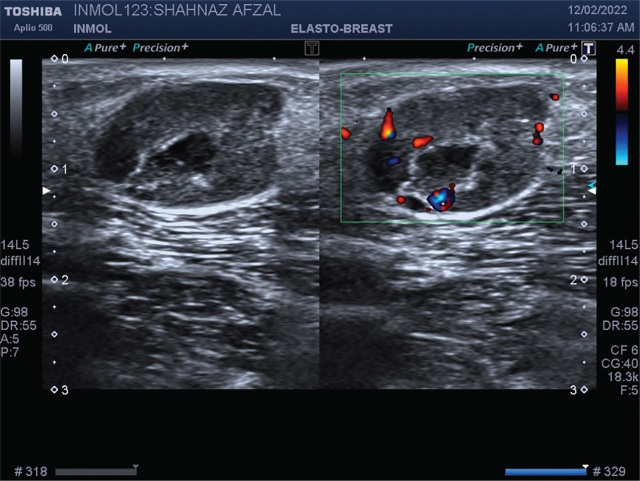
USG of cutaneous nodule revealed a 20 ×12 mm lobulated heterogeneous echogenicity lesion with internal necrotic changes in the subcutaneous plane of right axillary region, showing significant vascularity on color Doppler evaluation. USG, ultrasonography.

One month after the completion of treatment, the patient started to notice painful nodules in the bilateral axillary regions. For further evaluation, she was advised USG of both breasts and axillae. USG was performed using high-frequency linear transducer of TOSHIBA Aplio 500 that revealed a 20 × 12 mm lobulated heterogeneous echogenicity lesion with internal necrotic changes in the subcutaneous plane of right axillary region beneath the palpable tender nodular swelling, showing significant vascularity on color Doppler evaluation (**Figure 2**). A similar lesion measuring 12 × 10 mm was noted in the subcutaneous plane posterior to the right posterior axillary fold. In addition, a well-defined hypoechoic lesion measuring 7.2 × 4 mm was noted in the subcutaneous plane posterior to the left posterior axillary fold. The patient was advised histopathological correlation. Both breasts were unremarkable.

**Figure 3.**
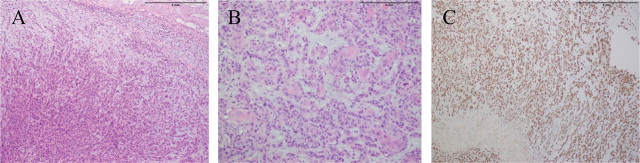
Histological images **(A, B)** show a cutaneous nodule infiltrated by a malignant neoplasm composed of sheets and nests of pleomorphic tumor cells with moderate cytoplasm and vesicular nuclei having prominent nucleoli. Foci of keratinization are also noted. **(C)** P63 immunostain nuclear staining highlights these atypical squamous cells.

The patient underwent excision biopsy of the larger lesion on the right side. Formalin preserved nodular fragment measuring 20 × 15 × 12 mm revealed nests and sheets of polygonal cells with hyperchromatic nuclei and moderate eosinophilic cytoplasm. Brisk mitosis and focal areas of keratinization were present. P63 immunostain nuclear staining highlighted atypical squamous cells. Findings were in favor of metastatic squamous cell carcinoma (**Figure 3**). A total of 6 cycles of Paclitaxel (240 mg) were planned as second-line chemotherapy.

## Discussion

Head-and-neck squamous cell carcinoma having cutaneous metastasis is rarely reported in the literature. Here, we report a case of relapse of squamous cell carcinoma of the hypopharynx which metastasized to the skin of the upper trunk region.

Among HNSCCs, hypopharynx is the most common primary to have distant metastasis followed by the anterior aspect and base of tongue. Distant cutaneous metastasis is, however, still very uncommon. The treatment plan regarding HNSCCs is beneficial at the locoregional control of disease but seldom fruitful at the metastatic sites. The risk of distant metastasis is increased by extracapsular spread from the lymph node. Aberrant and new lymphatics open up after neck dissection and radiotherapy, which may explain the concept of development of cutaneous metastasis [3].

Cutaneous metastasis is further subdivided into loco-regional and distant involvement. Loco-regional cutaneous metastasis results from lymphatic dissemination, iatrogenic implantation, or direct contiguous spread. In contrast, hematogenous dissemination is responsible for distant cutaneous metastasis.

The time of occurrence of cutaneous metastasis is also variable, ranging from 36 months to decades after the initial diagnosis. They have widespread clinical appearances and can resemble almost any dermatological condition, so a high level of suspicion is required by the clinician's eye regarding unnecessary delay in diagnosis. Classically, they are presented as erythematous, firm, painless nodules. Other common patterns include hemangioma-like, herpetiform, erysipelas-like, pyogenic, granuloma-like, morphea-like, bullous, and zosteriform lesions. However, there is no association between clinical presentation of skin lesion and tumor type. About 45% of skin lesions are not considered suspicious for metastasis initially. The diagnosis largely depends upon histopathological correlation.

The prevalence and type of primary cancer and frequency of occurrence of skin metastasis are very much related with the highest prevalence of cutaneous metastasis recorded in lung and breast cancer patients.

Schultz and Schwartz reported the first ever case of cutaneous metastatic deposits in hypopharyngeal carcinoma in 1985 [2].

The incidence of distant metastasis in more in hypopharyngeal and subglottic tumors as compared with glottic or supraglottic primaries.

Cutaneous metastasis to nasal tip in the setting of HNSCC is a rare event. A study conducted by Lopes Alexandre et al. [4] showed the development of cutaneous nasal tip metastasis in a patient of SCC of hypopharynx who had undergone pharyngolaryngectomy and adjuvant chemoradiotherapy 10 months ago. As patient was already receiving palliative treatment due to disease progression, so no significant change was made in the treatment plan. This scenario of “Clown Nose” is an example of rare location for cutaneous metastasis. Only 4 cases of nasal tip cutaneous metastasis secondary to HNSCC have been reported in literature. Out of these, 2 were from hypopharyngeal primary and 2 from the larynx. Araghi et al. [5] reported a rare case of multiple eruptive keratoacanthoma-like skin lesions in an area previously irradiated for laryngeal squamous cell carcinoma.

The median time of occurrence for cutaneous metastasis to develop is 6 months with about 90% mortality rate within 16 months of diagnosis. Two or more cervical nodal metastasis or extracapsular spread from involved cervical node closely relates with the development of cutaneous metastasis. Disease stage, however, does not predict the occurrence of cutaneous metastasis. The presence of heavy dermal component having no association with epidermis helps in distinguishing cutaneous metastasis from primary tumors of skin [6].

Metastatic cutaneous lesions are usually detected by patient observation, physical examination, USG, CT scan, or PET-CT. These modalities are helpful in assessment of the site and extent of spread of metastasis, either loco-regional or distant. Response to treatment is also dependent on radiological imaging. A recent advancement in this field is indocyanine green fluorescence angiography (ICG-FA), but some aspects of this modality are still unknown and under study [7].

In a meta-analysis conducted by Chiesa-Estomba et al. [8] factors contributing to development of cutaneous metastasis include hypopharyngeal site, lymph node size greater than 6 cm, poorly differentiated carcinoma on histopathology, and failure of loco-regional spread control.

Owing to advanced stage (III or IV) at presentation in 60%–80% patients, hypopharyngeal carcinoma is difficult to treat. The presence of cervical lymphadenopathy accounts for poor prognosis. Distant metastasis predominantly occurs in the liver, lung, bone, and mediastinal lymph nodes. In addition, nutritional and breathing difficulties pose a significant problem [9].

Patients of head and neck carcinomas require percutaneous endoscopic gastrostomy (PEG) tube for enteral nutrition owing to its minimally invasive protocol. The purpose is to provide hydration and nutritional support to the patient secondary to pharyngeal obstruction or effects of chemoradiotherapy. A study conducted by Greaves [10] showed HNSCC metastasis at PEG site with the reported rate of metastasis at stromal sites to be about 0.5%–1%.

A meta-analysis was conducted by Siu et al. [11] on 121 studies to look for the rates of gastrostomy site metastasis in patients with aero-digestive tract malignancies. Pooled analysis revealed the gastrostomy site metastasis rate to be 0.5%. The subgroup analysis showed the stomal site metastasis rate to be 0.29% (95% CI, 0.15%–0.55%) with the push technique and 0.56% (95% CI, 0.40%–0.79%) with the pull technique. The average time of development of stomal site metastasis from the time of placement of gastrostomy tube was 8 ± 5 months. This emphasizes the need for regular assessment of the gastrostomy site in hypopharyngeal carcinoma patients as well.

Development of cutaneous metastasis at different sites has been reported in different studies within 1 year of chemo and radiotherapy. Cutaneous metastasis carries a poor prognosis with palliative as the main treatment option [3].

Surgical excision or chemoradiotherapy treatment options largely depend on clinical perspectives of the disease process [6].

In short, the literature reports rare occurrence of distant cutaneous metastasis from HNSCC. If a new skin lesion is encountered in an HNSCC patient, it should have a high index of suspicion and he/she should undergo detailed history and complete physical examination and USG. The patient is to be followed vigilantly for early detection as cutaneous metastasis carries a poor prognosis and median survival.
